# Characterizing Urine and Sediment in Individuals with Lower Urinary Tract Dysfunction Utilizing Intermittent Catheters

**DOI:** 10.3390/jcm14238485

**Published:** 2025-11-29

**Authors:** Per Bagi, Christina Kruuse, Christian Forman, Betina Suldvart, Lotte Jacobsen, Marcio Augusto Averbeck, Michael Kennelly, Nikesh Thiruchelvam, Emmanuel Chartier-Kastler, Charalampos Konstantinidis, Andrei Krassioukov, Lene Feldskov Nielsen

**Affiliations:** 1 Department of Urology, Centre for Cancer and Organ Diseases, Copenhagen University Hospital Rigshospitalet, 2100 Copenhagen, Denmark; 2 Department of Brain and Spinal Cord Injury, Neuroscience Center, Copenhagen University Hospital Rigshospitalet, 2600 Glostrup, Denmark; 3 Coloplast A/S, 3050 Humlebæk, Denmark; 4 Moinhos de Vento Hospital, Porto Alegre 90560-032, Brazil; 5 Department of Urology, Carolinas Medical Center, Charlotte, NC 28203, USA; 6 Department of Urology, Cambridge University Hospitals NHS Trust, Cambridge CB2 0QQ, UK; 7 Department of Urology, Sorbonne Université, Academic Hospital Pitié Salpétrière, 75013 Paris, France; 8 Urology & Neurourology Unit, National Rehabilitation Center, 13122 Athens, Greece; 9 Division of Physical Medicine and Rehabilitation, Faculty of Medicine, The University of British Columbia, Vancouver, BC V5Z 2G9, Canada

**Keywords:** urinalysis, urinary sediment analysis, intermittent catheterization, lower urinary tract dysfunction

## Abstract

**Background/Objectives**: To perform a physicochemical characterization of urine and sediment in intermittent catheterization (IC) users and evaluate the impact of IC with micro-hole zone catheters (MHZC) and conventional two-eyelet catheters (CEC). **Methods:** Analysis of anonymized urine samples collected from four IC user groups with lower urinary tract dysfunction (LUTD): Newly diagnosed individuals with spinal cord injury (SCI) from an inpatient SCI clinic (A), and community-based IC users with SCI (B), multiple sclerosis (MS) (C), or other conditions than SCI or MS (D). Urine analysis included physicochemical properties, bacterial load, and sediment size, both after collection and following passage through MHZC and CEC. **Results**: Urine samples from 53 participants were analyzed (groups A: 11, B: 11, C: 9, D: 22). The physicochemical properties of urine were similar to reference values despite the prevalence of bacteriuria ranging from 54.5% to 77.3%. The median [99th percentile] sediment size in the total group was 8.6 µm [50.7 µm] and 8.5 µm [54.1 µm] for group A, 9.2 µm [40.3 µm] for group B, 7.9 µm [48.3 µm] for group C, and 8.9 µm [50.3 µm] for group D. Following catheter passage, the median sediment size for the total group was 8.9 µm with the MHZC and 8.9 µm with the CEC. **Conclusions**: This two-part study initially presented a novel approach to characterizing urine samples, including sediment from IC users, and, thereafter, an in vitro experiment using the samples to test sediment passage through MHZC and CEC. The results indicated similar urine properties and sediment sizes across groups and did not suggest differences or issues relating to urine and sediment passage through these IC technologies for these groups.

## 1. Introduction

For individuals with lower urinary tract dysfunction (LUTD), intermittent catheterization (IC) is generally considered to be the best bladder management option for minimizing the risk of urological complications [1]. A key quality for any bladder management option is whether the user is able to empty their bladder efficiently, leaving minimal residual urine in the bladder, as residual urine is a recognized risk factor for urinary tract infection (UTI) [2,3,4]. IC is well-documented as an efficient method for bladder emptying that ensures minimal residual urine [5], but conventional two-eyelet catheters (CEC) may also be associated with episodes of compromised bladder drainage due to mucosal suction and flow-stops [6], which newer IC technology, such as the micro-hole zone catheter (MHZC), is designed to avoid [7]. Efficient bladder emptying also includes clearing the particles found in urine as sediment. Some evidence already indicates that switching from indwelling catheterization to frequent IC can reduce the amount of urinary sediment in the bladder in catheter users with neurogenic LUTD [8]. Nevertheless, the ability of both MHZCs and CECs to drain urine with sediment requires proper assessment, including evaluation of the physicochemical and biological properties of the urine and microscopic investigation of the urinary sediment [9,10,11,12].

The physicochemical properties of urine and their variation as a function of health status, metabolism, and lifestyle-related factors are generally well-described for healthy individuals, including reference intervals on parameters such as pH, viscosity, osmolality, density, and conductivity [13]. Furthermore, microscopic investigation of urinary sediment [14] has cataloged numerous different types of sediment, with some of the most common types including red and white blood cells, epithelial cells, lipids, urinary casts, crystals, and microorganisms [12,15,16,17,18]. Most of these microscopic particles are not uncommon findings in smaller quantities as sediment in urine samples from healthy individuals but can indicate a potential health issue above certain thresholds, for example, in the case of certain types of red and white blood cells, and epithelial cells [13].

Similarly, several types of common minerals in urine can form microscopic crystals that can be found as sediment in healthy individuals, but increased levels of crystalluria also might stem from metabolic changes. These can include exogenous causes such as increased exposure to certain high mineral content foods or drugs, be caused by endogenous conditions affecting mineral excretion and crystallization rates, or be associated with a range of kidney disorders, including nephropathy or nephrolithiasis [16,18]. These can provide important diagnostic information about stone-forming diseases, in combination with physicochemical tests [19]. While less frequently observed, notable work has also gone into describing the range of larger, aggregated, and cylindrical particles, such as casts, and investigating their association with health conditions [18,20].

Although considerable knowledge exists to understand the function of urine sediment as a general health indicator in individuals with intact urinary tract function, its relation to IC remains underexplored, as no studies have investigated these physicochemical characteristics of urine and urinary sediment in individuals with LUTD using IC. This includes whether or not urine sediment could have consequences for the use of IC and whether LUTD, and whether or not the use of IC for bladder emptying is associated with differences in the urine sediment content.

To support the continued advancement of bladder health in IC users with LUTD, it is essential to better understand their urinary sediment. Specifically, whether sediment is predominantly characterized by particle sizes similar to the well-described size distribution of the cells and crystals generally expressed in urinary sediment, which are much smaller than the 400 µm micro-hole size of the MHZC [15,18,21], or to what extent any other types of sediment are also present. To help close this knowledge gap and better understand the urine and urinary sediment drained during catheterization, the study aimed to perform a physicochemical analysis of freshly catheterized urine from four groups, including groups that were exclusively comprising individuals using IC because of the common causes of neurogenic LUTD, namely spinal cord injury (SCI) and multiple sclerosis (MS), as well as a group including a mix of causes of LUTD, both neurogenic and non-neurogenic. This included a descriptive analysis of the size distribution of urinary sediment and an evaluation of whether the passage of sediment through the catheter is affected by different IC technologies.

## 2. Materials and Methods

### 2.1. Study Design

This investigation collected anonymized urine from a broad range of IC users, including participants from four different groups. The inclusion and exclusion criteria for each group are listed in Table 1. Informed consent was obtained from all participants involved in the study.

### 2.2. Sample Collection and Test Procedures

**Figure 1:**
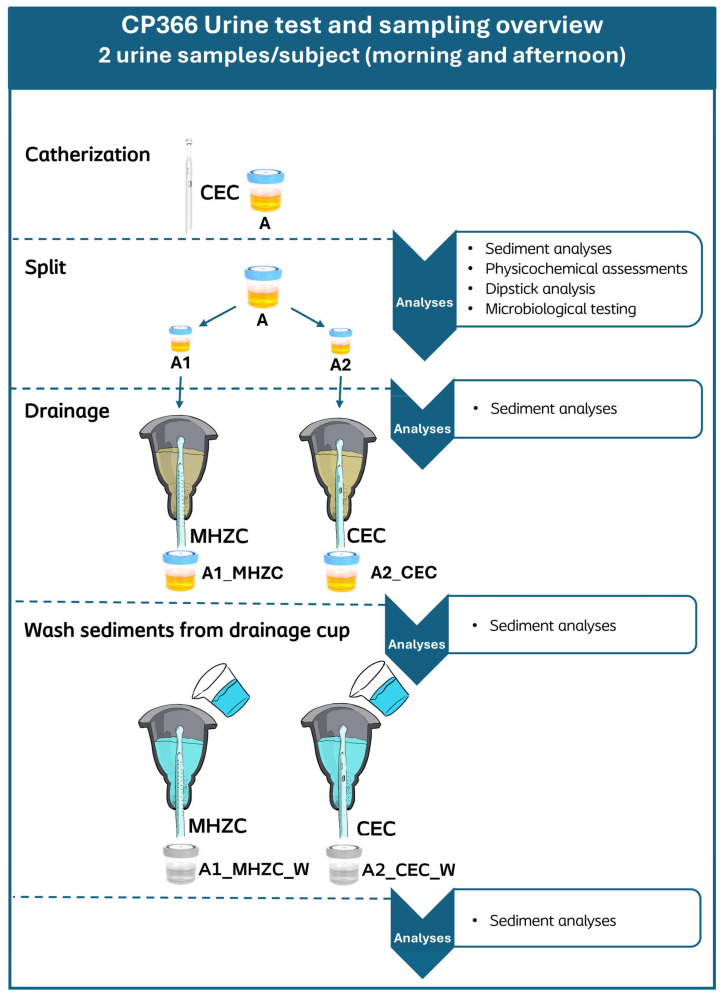
Urine sample processing pipeline and applied analyses at each stage of processing. Immediately after collection, sediment analysis, physicochemical analyses, and dipstick analysis were performed on the drained urine sample (A). Afterwards, sample A was split into two 50 mL aliquots (A1 and A2) and analyzed for sediment. These aliquots were then transferred to a cup, from which the urine could be drained with either an MHZC (A1_MHZC) or a CEC (A2_CEC), respectively, before being analyzed for sediment again. When the cup had been emptied, 50 mL of Milli-Q water was added to rinse and drain any remaining sediment, producing the aliquots A1_MHZC_W and A2_CEC_W, which were also analyzed for sediment. All aliquot results of both samples from each participant were included in the statistical analysis. MHZC = Micro-hole zone catheter. CEC = Conventional Eyelet Catheter.

Anonymized urine samples were collected in sterile containers by means of IC with a CEC, Charrière size (CH) ≥ 12. Participants were instructed to clean the peri-urethral area prior to obtaining the samples, according to the information for the users (IFU) of the CEC used. Two samples were collected from each participant, with a minimum of 2 h between the two catheterizations, and were separately passed through the whole process. Some participants from group A, who had recently begun self-catheterization and could not yet reliably perform a full emptying of the bladder according to best practice guidelines, were instead catheterized by a healthcare professional (HCP), ensuring both the health of the subject’s urinary tract and the standardization of the urine sample. For these same reasons, all self-catheterizing subjects across the groups were asked to perform a full standard catheterization to achieve an empty bladder. Urine samples were kept at room temperature and under gentle stirring to ensure homogeneity of the particle dispersion from collection and until completion of all dipstick-, microbiological-, and sediment analyses. The testing procedure for each urine sample is presented in Figure 1.

### 2.3. Urine Sediment Analysis Methods

Sediment analysis was performed on all urine samples and aliquots by pumping the sample through a flow cell (oCelloScope, BioSense Solutions, Farum, Denmark) with 10 replicates captured per sample for analysis, including sediment size distribution through automated particle segmentation software (ParticleTech ApS, Farum, Denmark), counting and measurement of the maximum Feret diameter (FeretMax), i.e., the longest dimension of each sedimented particle in the urine. Identification was performed on particles with a minimum size of 2.5 µm, and an algorithm-based sediment classification based on particle opacity was applied to identify whether a urine sample contained both crystalline and non-crystalline components. This algorithm-based sediment classification was developed specifically for this study in collaboration with the software provider, training the algorithm by feeding manually labeled images of the most common sediment types. As this method is still exploratory, sediment classification was limited to two categories, crystalline and non-crystalline. Non-crystalline components, including cells, microorganisms, and other lipid- or protein-based biological aggregates, are referred to as organically composed sediment in this study, despite not covering all organic urinary compounds [22]. If the automated analysis identified a particle with a FeretMax size over 400 µm in the freshly collected urine sample (A), indicating the longest dimension of that particle to be larger than a single hole of the MHZC, an additional manual assessment and description of particle structure and composition was performed. All subsequent aliquots were described by size distribution and particle count/mL.

### 2.4. Physicochemical and Microbiological Analysis

The physicochemical characteristics measured included the urine pH (SI analytics Lab 845, Xylem, Weilheim, Germany), the osmolality (Osmomat 3000, Gonotec^®^, MD Scientific ApS, Aarhus, Denmark), conductivity (Seven Excellence Multiparameter, Mettler Toledo, Glostrup, Denmark), viscosity (DMA 4100 M, Anton Paar Nordic AB, Ballerup, Denmark), and density (DMA 4100 M, Anton Paar Nordic AB, Ballerup, Denmark). All urine samples (A) were tested by means of a urine dipstick (Multistix^®^ 10 SG reagent strips, Siemens Healthineers, Erlangen, Germany) and analyzed by means of a Siemens Clinitek Status + Analyzer, for the following parameters: protein, hematuria, leukocytes, nitrite, glucose, ketones, bilirubin, urobilinogen, specific gravity, and pH. Both viscosity and density were measured at 37 °C, whereas all other physicochemical parameters were measured at room temperature. Microbiological testing was conducted with each of the A samples on chromogenic agar plates (Flexicult ID plates, SSI Diagnostica, Hilleroed, Denmark), which were incubated bottom up at 35 °C for a total of 48 h. According to the corresponding instructions, identification of the bacterial strain was performed after the first 18 h to 24 h, based on the colonies’ colors and sizes, and a colony count (CFU) was also performed. The agar plates were then incubated for the remaining time, after which the total bacterial count was assessed. Any presence of bacteria in the urine samples was noted, with the presence of bacteriuria defined as a CFU count/mL of 103 or higher, according to the European Association of Urology (EAU) guidelines on bacteriuria in catheter users [23].

### 2.5. Statistical Analysis

The statistical analysis was performed using SAS (Enterprise Guide version 7.3, SAS Institute Inc., Cary, NC, USA). Due to the study’s observational design and exploratory aim to describe the range of urinary sediment sizes rather than any average values, only descriptive statistics were applied to the sediment sizes with no formal statistical comparisons between groups. With the current lack of scientific literature for comparison, it would not be possible to estimate a minimal clinically important difference for this variable, and to focus on *p*-values would risk obscuring the more relevant clinical discussion of the implications of sediment sizes for LUTD and IC use. The descriptive statistics used to describe the size distributions included the N, median, and 99th percentile. However, particle counts/mL, physicochemical properties, and bacterial counts were described by their mean and standard deviation. For discrete variables such as the dipstick measurements and microbiological testing for bacterial strains and bacteriuria, descriptive statistics are presented with N and percentage. The N denoted the number of participants contributing with non-missing data.

## 3. Results

### 3.1. Urine Characteristics

The study included 53 participants (14 female, 39 male) with a mean (SD) age of 69.2 (14.9) years. Groups A, B, C, and D included 11, 11, 9, and 22 participants, respectively. Physicochemical results are reported in Table 2, with results presented for each group. Results of the microbiological investigation, including identification of bacterial strains and count of CFU for each urine sample for each group, are shown in Table 3. The results of dipstick testing the collected urine samples are presented in Table S1 in the Supplemental Materials.

### 3.2. Urine Sediment Analysis

**Figure 2:**
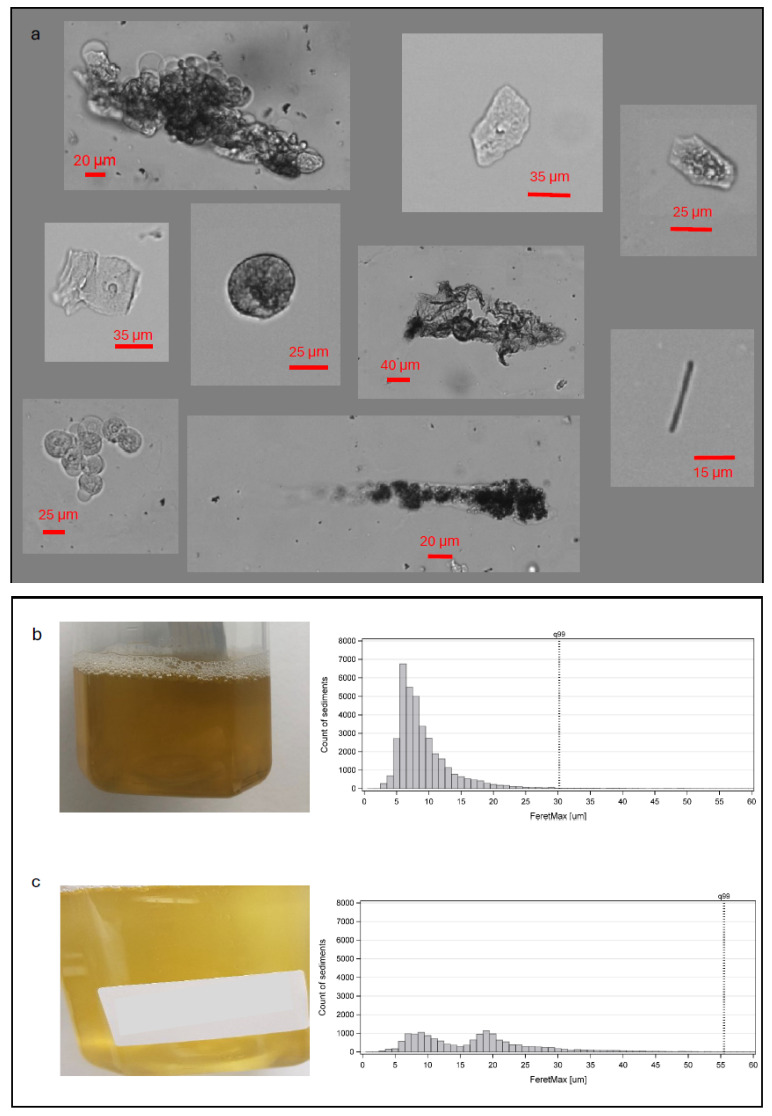
(**a**) Characteristic examples of individual particles identified as urinary sediment, including examples of different cell types, a rod-shaped crystal (bottom right corner), and examples of aggregated clusters of particles. Each particle was analyzed for its length along its longest dimension. (**b**) One example of an individual urine sample with apparent high turbidity and its corresponding size distribution histogram peaking around the 5–10 µm sizes. (**c**) A urine sample from another participant with apparent lower turbidity, where the majority of sediment sizes fall within the 5–25 µm range.

The sediment analysis identified a total of 3,198,427 individual particles in the collected urine samples. Examples of some of the different types of sediment identified are presented in Figure 2. The identified sediment types in the urine samples included both organic and crystalline sediment in 20 of 22 samples (90.9%) in group A, 15 of 22 samples (68.2%) in group B, 13 of 18 samples (72.2%) in group C, and 38 of 44 samples (86.4%) in group D. The remaining samples of all four groups were classified as containing exclusively organic sediment, with no samples containing exclusively crystalline sediment.

### 3.3. Sediment Size Distribution

**Figure 3:**
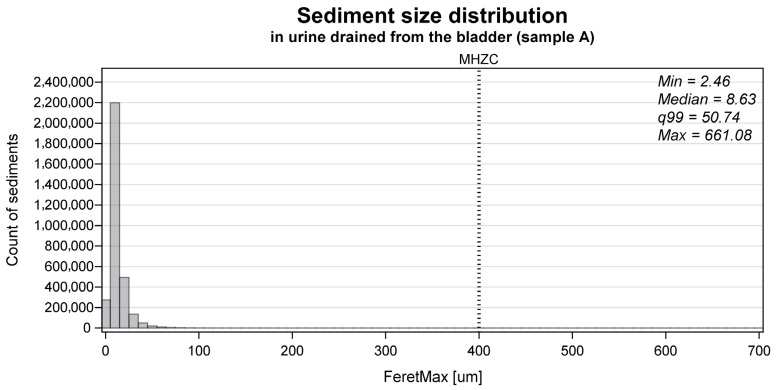
The size distribution of all particles identified as sediment in the collected urine sample (A) (n = 3,198,427) measured as the FeretMax size. The dotted vertical line tagged ‘MHZC’ represents the 400 μm hole size of the micro-hole zone catheter.

The median sediment size in the total group was 8.6 µm, with the 99th percentile being 50.7 µm. The size distribution of all particles identified in the urinary sediment of the A samples in the total group is presented in Figure 3. For the individual groups, the median [99th percentile] size was 8.5 µm [54.1 µm] for group A, 9.2 µm [40.3 µm] for group B, 7.9 µm [48.3 µm] for group C, and 8.9 µm [50.3 µm] for group D. The mean (SD) particles/mL in the urinary sediment was 2.8 × 10^6^ particles/mL (2.8 × 10^6^ particles/mL) for group A, 3.7 × 10_5_ particles/mL (4.9 × 10^5^ particles/mL) for group B, 9.4 × 10^5^ particles/mL (1.0 × 10^6^ particles/mL), and 2.4 × 10^6^ particles/mL (2.6 × 10^6^ particles/mL) for group D.

**Figure 4:**
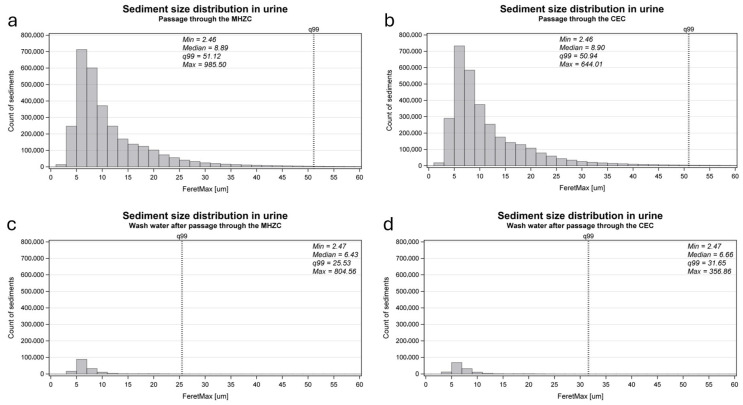
The size distribution of urinary sediment in the aliquots passed through a MHZC (‘A1_MHZC’, (**a**)) or a CEC (‘A2_CEC’, (**b**)) and in the water applied to rinse the drainage cups afterwards (‘A1_MHZC_W’ and ‘A2_CEC_W’, (**c**,**d**)). To achieve a higher resolution of the size distribution of the majority of sediment sizes, the graphs do not include the full range of sizes; however, no graphs include less than 99% of the observed sizes (indicated by the vertical dotted line marked q99, specifying the 99th percentile).

The sediment size distribution in the urine aliquots, which had passed through either an MHZC or a CEC, and the water used to rinse the drainage cup with the MHZC and with the CEC are presented in Figure 4. The median sediment size of the total group after catheter passage was 8.9 µm with the MHZC and 8.9 µm with the CEC. In the water used to rinse the drainage cup, the median sediment size left behind was 6.4 µm for the MHZC and 6.7 µm for the CEC.

### 3.4. Visual Assessment of Largest Particles

**Figure 5:**
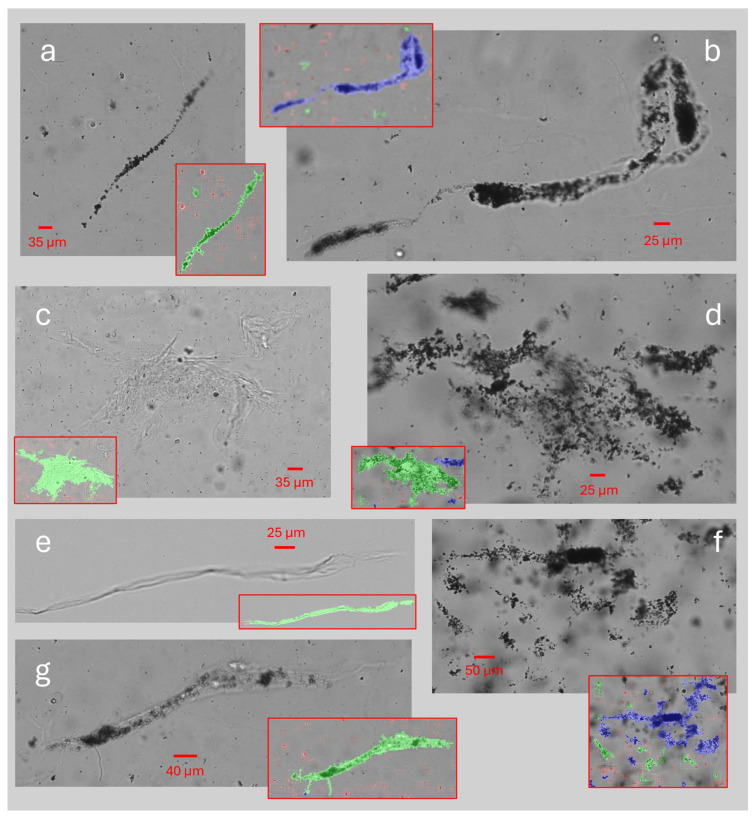
Examples of large particles (range 400–661 μm) identified as sediment and segmented by the sediment analysis as a single compound. Overlayed images in red frames indicate how the particles in the corresponding image were segmented into separate compounds: (**a**–**e**) were found in the freshly drained urine samples (sample A); (**f**,**g**) were found in the aliquots which had been passed through an MHZC (A1_MHZC).

A total of 10 of the 3,198,427 (~0.0003%) particles identified as sediment in the freshly drained urine samples A had a FeretMax diameter of size 400 μm or larger. These were identified in the urine samples from four different participants, two belonging to group A, one to group C, and one to group D, and representative examples of the different particle types are presented in Figure 5. The maximal size measured in the freshly drained urine samples was 661.1 µm [Figure 5b]. When testing the aliquots after catheter passage, seven particles larger than 400 μm were identified in the aliquots passed through the MHZC, while four particles were identified in the aliquots passed through the CEC.

## 4. Discussion

### 4.1. Characteristics of Urine and Sediment in IC Users

The aim of this study was to perform a detailed characterization of freshly drained urine from a wide group of IC users, including the size and amount of urinary sediment, the physicochemical properties of the urine, and the presence of bacteria. The study identified and analyzed almost 3.2 million individual particles of sediment in the freshly captured urine samples from 53 IC users. The sediment size distribution showed that 99% of urinary sediment sizes were smaller than 50.7 µm, with a median size of 8.6 µm. This observation, which has not previously been described, supports that most of the particles constituting urinary sediment in IC users fall within the well-established sizes of cells and other components, such as those most commonly observed in urine in general, irrespective of health status [13,15,18].

The median size of the urinary sediment only varied by 1.3 µm in the four different groups, which suggested that neither pathology, group, nor duration of spinal cord injury or intermittent catheter use had a distinctly different sediment size distribution. No formal statistical analysis was applied to compare the sizes of the sediment between the four groups. This was chosen due to the novelty of the automated quantitative sediment analysis, for which the knowledge of minimal clinically important differences or general reference values for size distributions in different populations remains sparse. It is possible that the large number of data points collected during the automated sediment analysis could result in statistically significant *p*-values, even with the small observed differences between the groups’ median values, down to a single µm or less, which are unlikely to be clinically relevant. Future studies should aim to apply this quantitative sediment analysis in larger cohort studies to validate the findings of this study across the patient etiologies and begin to understand clinically important differences in sediment size distributions. The focus of the present article is, therefore, instead to discuss how the observed sediment size distributions relate to LUTD and IC use and how automated quantitative sediment analysis can provide clinically relevant information to the HCPs advising on IC use.

To capture a comprehensive picture of the characteristics of urine in IC users, a representative selection of IC user etiologies was included in the study, both with neurogenic and non-neurogenic LUTDs, and new and experienced users of IC. Individuals with a recent history of stone-forming disease or with a neo-bladder or augmented bladder were excluded from participation. This was performed to describe the general IC user population and avoid interference by other pathologies of the urinary tract associated with large excretions of a specific compound through the urinary tract. The inclusion of two separate SCI groups can further have contributed to a higher number of male participants, as SCI is more prevalent in males [24], but this is not likely to have markedly impacted the sediment distribution. Our results show that the physicochemical properties of urine in the groups in focus were not dissimilar to those of the general population [13]. Despite the diversity of pathologies causing the need for IC, the physicochemical properties of urine were comparable between the groups and within normal urine reference values for pH [13], with the viscosity being similar or slightly thinner than reference values [25], and the osmolality similarly ranging from within normal values to slight dilution [26]. References on the density of urine cite relative density values at room temperature rather than absolute density [13], which explains why this study saw slightly lower absolute density results, being measured at 37 °C. As conductivity relates to the concentration of salts in the urine [13], the results could suggest a slightly higher concentration in the urine of the newly injured and still hospitalized individuals with SCI. However, no clear reference range exists, and all four groups fall well within the range of values reported in other studies [27]. A higher urine salt content in the hospitalized individuals with SCI could likely be explained by the bone demineralization and hypercalciuria often seen for months following an SCI [28]. The microbiological investigation of the study revealed the presence of different bacterial strains in the urine samples, with *Escherichia coli* and *Enterococcus faecalis* being the most common overall. Almost all included IC users had some presence of bacteria in their urine, with approximately two-thirds of them indicated to have bacteriuria by EAU standards in at least one of their urine samples [23]. Such levels of bacteriuria are common in many populations and are comparable to those reported earlier for IC users, which is a frequent finding even in the absence of symptoms of UTI [29]. Any treatment hereof in the absence of UTI symptoms is generally advised against [4,23,30]. These results indicate that this study’s groups covered a representative selection of IC users, supporting the generalizability of the results.

### 4.2. Sediment Drainage by Intermittent Catheters

To further explore the dynamics of how sediment is passed during IC, this study set up an in vitro experiment, testing what happens to the urinary sediment size distribution after passage through two different types of intermittent catheters, the MHZC and CEC, presented in Figure 1. Following catheter passage, the median and 99th percentile sizes were similar when compared between the MHZC and CEC, and these were both similar to the initially collected sample. These results suggested that the sediment passes through the two types of intermittent catheters to the same extent. Some sediment remained in the drainage cup after completion for both types of catheters, although the vast majority of sediment passed through the catheters with both the MHZC and the CEC. The remaining sediment in the cup had smaller median and 99th percentile sizes and is likely to have adhered to the sides of the container during the test, after which it was drained without issue as part of the water used to rinse it.

A challenge when performing automated segmentation of microscopic particles is to analyze clusters of particles that are either loosely bound together, aggregated in a larger structure, or simply positioned too closely together to distinguish their borders. In urinary sediment, all these clusters are likely to be present. Several types of sediment, including lipids, granulocytes, and various types of epithelial cells, are known to appear both individually and as clusters [13]. Urinary casts constitute the most likely findings of long aggregated structures and can contain a variety of sediment types, including cells, bacteria, and occasionally crystals, encapsulated within a protein matrix [18]. Finally, some smaller types of sediment, including bacteria or small crystals, could, in some cases, be numerous and closely positioned enough that they could appear connected [15].

Of almost 3.2 million individual particles of sediment studied in the freshly drained urine samples, 10 were larger than 400 µm when measured along their longest diameter. The particle images were retrieved for visual assessment of their shape and structure. Some images, both in Figure 2a and Figure 5, clearly identified large components of the sediment with elongated and variable shapes, bent and wrinkled edges, containing aggregations of various types of sediment within the matrix, fitting the description of urinary casts [13,18]. Previous studies of urinary casts have also observed similar lengths as this study [20]. Their shape most likely allows them to pass through catheter eyelets easily, further enabled by their composition of aggregated smaller components in a protein matrix, possibly allowing some structural flexibility. The microscopic analysis in this study could not determine the origin of specific particles and, as such, could not with certainty identify casts. To better understand the presence of specific types of sediment in the urine of IC users, future studies including more advanced imaging methods would be required. Other examples of large particle sizes could be multiple separate elements appearing connected because of their close proximity, also exemplified in Figure 5. In these images, many similar-looking particles appear to form one aggregate, although no encapsulating structure is visible, and the particles in some places appear only closely positioned but not structurally linked. It is plausible that some of these particles do not pass through the catheter eyelets as a combined structure. Instead, they might appear as a single structure in the image, resulting either from specific physicochemical properties of the urine or from the flow dynamics of the sample through the equipment used for measurement. Finally, various potential contaminants, such as fibers from the participants’ clothing, are also described and occasionally mistaken for other types of urinary sediment, including casts [15]. The study aimed to perform sediment analysis as quickly after sample collection as possible and with minimal handling and influence on the components to ensure the integrity of the cells and prevent physicochemical changes related to sample storage. Consequently, it was not possible to confirm whether all identified larger particles could be considered urinary sediment originating from the bladder.

### 4.3. Future Directions

The results of the present study contribute to the understanding of the urine and sediment dynamics involved in efficient bladder emptying, which is key to ensuring bladder health in individuals with LUTD relying on IC, but much remains to be understood about optimal bladder emptying by IC. This study collected real urine samples with natural sediment from IC users to characterize and then use them for conducting in vitro testing of the flow and passage of urine and sediment through catheter eyelets. This was performed to measure the sediment content of the urine both before and after the in vitro setup, testing which changes occurred as a result of catheter passage. However, bladder emptying also relies on real-world parameters such as the flow dynamics of the urine, which help carry the sediment out from the bladder. Sediment is likely to collect towards the bottom of the bladder, close to the bladder neck, meaning that catheter eyelets positioned further from the bladder neck could risk less efficient sediment drainage, even if repositioning the catheter closer to the bladder neck towards the end of catheterization, as the ratio of residual urine to sediment may not be sufficient to flush out the sediment. Future in vivo studies should investigate how urine flow dynamics during catheterization affect the ability of the urine to carry sediment from the bladder.

## 5. Conclusions

This study is the first of its kind to systematically characterize the urine and quantify the urinary sediment in IC users. The urine characterization indicated that the sediment in IC users’ urine is predominantly made up of smaller sized particles, similar to the sizes of the cells and other components commonly found in urine as sediment, and that the mean physicochemical properties of the urine are not different from normal reference values, despite the common observation of urinary sediment and the well-established presence of bacteria in the urine. A total of 99% of sediment sizes had the longest dimension below 50.7 µm, and only approximately 0.0003% had the longest dimension larger than 400 µm, constituting the hole size of the MHZC. The following in vitro experiment did not identify any differences between sediment passage through the MHZC and the CEC or any issues relating to the passage of urine and urinary sediments through these IC technologies for the included groups of IC users. With the observations of the study collectively, particularly the presented general size distribution of sediment and physicochemical properties of IC users’ urine, no concerns were identified regarding the ability of either of the intermittent catheters to pass urine and urinary sediment in this selection of IC users. Considering the range of important catheterization-related factors to address when supporting IC users to find their best solution, this study indicated that urinary sediments very rarely contain elements of a size that could challenge catheterization using IC. Future in vivo studies should further investigate real-world bladder-emptying parameters, including sediment removal, to understand the influence of catheter design on urine and sediment flow and support patient education.

## Figures and Tables

**Table 1:** The inclusion and exclusion criteria of the study.

Inclusion criteria	
Group A	Group B, C, and D
Has given written informed consent	Has given written informed consent
Has full legal capacity	Has full legal capacity
Has been diagnosed with a SCI within the last 3 months	For B: has been diagnosed with SCI for more than 3 months For C: has been diagnosed with MS For D: has not been diagnosed with SCI or MS but is using IC for any other reason
Is hospitalized at a rehabilitation center/hospital	Has been using IC daily (but not necessarily exclusively) for bladder emptying, for at least 3 months
Is using IC as a primary method for bladder emptying	Can perform self-catheterization
Can use a 2-eyelet catheter, minimum size CH12 for IC	Is using a two-eyelet catheter, minimum size CH12, for IC
**Exclusion criteria (all groups)**	
Has previously participated in this study	
Has a recent (within 2 years) history of stone formation (kidney stones or bladder stones)	
Has a Neobladder or an augmented bladder	

**Table 2:** Results of the physicochemical characteristics of urine for each group presented with mean and standard deviation (SD).

Physicochemical Characteristics	Group A, Mean (SD) N = 11	Group B, Mean (SD) N = 11	Group C, Mean (SD) N = 9	Group D, Mean (SD) N = 22	Total Mean (SD)
pH (pH meter)	6.26 (0.83)	6.54 (0.66)	6.59 (0.88)	5.96 (0.66)	6.25 (0.78)
Viscosity (mPa/s)	0.79 (0.08)	0.71 (0.02)	0.72 (0.03)	0.72 (0.03)	0.73 (0.05)
Density (G/cm^3^)	1.01 (0.01)	1.00 (0.01)	1.00 (0.01)	1.00 (0.01)	1.00 (0.01)
Osmolality (mOsm/Kg)	481 (205)	256 (133)	296 (204)	345 (161)	346 (188)
Conductivity (mS/cm)	12.38 (5.29)	8.21 (3.59)	8.23 (4.54)	9.69 (3.77)	9.70 (4.44)

**Table 3:** Overview of the presence of bacteriuria, the bacterial species identified in the urine samples, and the overall mean bacterial count for each of the four groups. The ‘n = ’ denotes the number of participants included in the group. * Bacterial presence was counted as the number of participants in whom the strain of bacteria was found in either of their two samples. ** The number of participants who had at least one of their urine samples showing >0 CFU/mL. *** Bacteriuria was defined in accordance with EAU guidelines, as the participant having at least one of their two collected urine samples showing CFU/mL counts of 10^3^ or higher [23].

Bacterial Strain *, Count of Positives (%)	Group A (n = 11)	Group B (n = 11)	Group C (n = 9)	Group D (n = 22)
*Escherichia coli*	6 (54.5)	6 (54.5)	4 (44.4)	10 (45.5)
*Klebsiella species*	4 (36.4)	1 (9.1)	1 (11.1)	4 (18.1)
*Enterobacter species*	0 (0.0)	1 (9.1)	0 (0.0)	0 (0.0)
*Proteus species*	0 (0.0)	0 (0.0)	0 (0.0)	1 (4.5)
*Proteus vulgaris*	0 (0.0)	0 (0.0)	0 (0.0)	0 (0.0)
*Pseudomonas aeruginosa*	0 (0.0)	1 (9.1)	0 (0.0)	4 (18.1)
*Enterococcus faecalis*	5 (45.5)	3 (27.3)	1 (11.1)	7 (31.8)
*Enterococcus faecium*	0 (0.0)	1 (9.1)	1 (11.1)	1 (4.5)
*Staphylococcus saprophyticus*	1 (9.1)	1 (9.1)	2 (22.2)	5 (22.7)
*Candida species*	1 (9.1)	5 (45.5)	4 (44.4)	3 (13.6)
*Other strains*	0 (0.0)	0 (0.0)	0 (0.0)	1 (4.5)
Bacteria identified in urine **	10 (90.9)	10 (90.9)	9 (100)	20 (90.9)
Bacteriuria ***, ≥10^3^ CFU/mL	6 (54.5)	7 (64.6)	4 (66.7)	17 (77.3)
Mean bacterial count, CFU/mL (SD)	2.4 × 10^7^ (4.1 × 10^7^)	8.2 × 10^4^ (2.2 × 10^5^)	9.3 × 10^6^ (1.7 × 10^7^)	2.1 × 10^7^ (3.7 × 10^7^)

## Data Availability

The data presented in this study are available upon reasonable request from the corresponding author.

## Supplementary Materials

The following supporting information can be downloaded at: https://www.mdpi.com/article/10.3390/jcm14238485/s1, Table S1: Urine dipstick test results.

## Abbreviations

The following abbreviations are used in this manuscript:

CEC	Conventional Eyelet Catheter
CH	Charrière
HCP	Healthcare Professional
IC	Intermittent Catheterisation
IFU	Information For Users
LUTD	Lower Urinary Tract Dysfunction
MHZC	Micro-Hole Zone Catheter
MS	Multiple Sclerosis
SCI	Spinal Cord Injury
SD	Standard Deviation
UTI	Urinary Tract Infection
