# Supplementary material for: Characterizing Urine and Sediment in Individuals with Lower Urinary Tract Dysfunction Utilizing Intermittent Catheters
Source: J Clin Med. 2025 Nov 29;14(23):8485. doi: 10.3390/jcm14238485 (PMC12693602; doi:10.3390/jcm14238485)
Supplement: Supplementary file 1 [file jcm-14-08485-s001.zip › jcm-3984859-supplementary.pdf]

Supplementary Materials

*Table S1: *Explanation of hematuria levels: Level 1: 0 erythrocytes/mL (non-hemolyzed), Level 2: 10 erythrocytes/mL (non-hemolyzed), Level 3: 80 erythrocytes/mL (non-hemolyzed), Level 4: 10 erythrocytes/mL (hemolyzed), Level 5: 25 erythrocytes/mL (hemolyzed), Level 6: 80 erythrocytes/mL (hemolyzed), Level 7: 200 erythrocytes/mL (hemolyzed). The specified number of samples (n) includes both samples that were collected from each subject.*

Urinary dipstick tests	Group A (n=22)	Group B (n=22)	Group C (n=18)	Group D (n=44)
**Protein, n (%)**				
>= 300 mg/dL	0 (0)	0 (0)	0 (0)	1 (2.3)
100 mg/dL	0 (0)	0 (0)	0 (0)	3 (6.8)
30 mg/dL	2 (9.1)	0 (0)	0 (0)	0 (0)
Negative	19 (86.4)	22 (100)	17 (94.4)	38 (86.4)
Trace	1 (4.5)	0 (0)	1 (5.6)	2 (4.5)
**Blood, n (%)***				
Level 1	12 (54.4)	19 (86.4)	15 (83.3)	24 (54.5)
Level 2	8 (36.4)	1 (4.5)	1 (5.6)	6 (13.6)
Level 3	0 (0)	1 (4.5)	1 (5.6)	2 (4.5)
Level 4	0 (0)	0 (0)	0 (0)	0 (0)
Level 5	2 (9.1)	1 (4.5)	1 (5.6)	3 (6.8)
Level 6	0 (0)	0 (0)	0 (0)	4 (9.1)
Level 7	0 (0)	0 (0)	0 (0)	5 (11.4)
**Leukocytes, n (%)**				
0	14 (63.6)	14 (63.6)	10 (55.6)	9 (20.5)
1+	0 (0.0)	3 (13.6)	4 (22.2)	10 (22.7)
2+	6 (27.3)	4 (18.2)	2 (11.1)	9 (20.5)
3+	0 (0.0)	1 (4.5)	2 (11.1)	9 (20.5)
4+	2 (9.1)	0 (0.0)	0 (0.0)	7 (15.9)
**Nitrites, n (%)**				
Positive	5 (22.7)	0 (0.0)	3 (16.7)	7 (15.9)
Negative	17 (77.3)	22 (100)	15 (83.3)	37 (84.1)

**Glucose, n (%)**				
100 mg/dL	3 (13.6)	0 (0.0)	0 (0.0)	1 (2.3)
500 mg/dL	1 (4.5)	0 (0.0)	0 (0.0)	0 (0.0)
>=1000 mg/dL	2 (9.1)	0 (0.0)	0 (0.0)	2 (4.5)
Negative	16 (72.7)	22 (100)	18 (100)	41 (93.2)
**Ketones, n (%)**				
15 mg/dL	1 (4.5)	0 (0.0)	0 (0.0)	2 (4.5)
40 mg/dL	1 (4.5)	0 (0.0)	0 (0.0)	0 (0.0)
Negative	20 (90.9)	22 (100)	18 (100)	42 (95.5)
**Bilirubin, n (%)**				
Positive	0 (0.0)	0 (0.0)	0 (0.0)	0 (0.0)
Negative	22 (100)	22 (100)	18 (100)	44 (100)
**Urobilinogen, n (%)**				
0.2 mg/dL	21 (95.5)	22 (100)	18 (100)	43 (97.7)
1.0 mg/dL	1 (4.5)	0 (0.0)	0 (0.0)	1 (2.3)
**Specific Gravity, n (%)**				
<= 1.005	1 (4.5)	3 (13.6)	0 (0.0)	0 (0.0)
1.010	3 (13.6)	4 (18.2)	5 (27.8)	17 (38.6)
1.015	6 (27.3)	8 (36.4)	8 (44.4)	10 (22.7)
1.020	7 (31.8)	4 (18.2)	3 (16.7)	7 (15.9)
1.025	3 (13.6)	2 (9.1)	2 (11.1)	5 (11.4)
>= 1.030	2 (9.1)	1 (4.5)	0 (0.0)	5 (11.4)
**pH**				
Mean (SD)	6.0 (0.7)	6.4 (0.9)	6.4 (0.8)	5.9 (0.7)
